# SARS-CoV-2 Seroprevalence Study in Pediatric Patients and Health Care Workers Using Multiplex Antibody Immunoassays

**DOI:** 10.3390/v14092039

**Published:** 2022-09-14

**Authors:** Esther Prados de la Torre, Ignacio Obando, Marta Vidal, Beatriz de Felipe, Ruth Aguilar, Luis Izquierdo, Carlo Carolis, Peter Olbrich, Ana Capilla-Miranda, Pau Serra, Pere Santamaria, Pilar Blanco-Lobo, Gemma Moncunill, Manuel J. Rodríguez-Ortega, Carlota Dobaño

**Affiliations:** 1 Departamento de Bioquímica y Biología Molecular, Campus de Excelencia Internacional CeiA3, Universidad de Córdoba, 14071 Córdoba, Spain; 2 Unidad de Pediatría, Sección de Infectología, Reumatología e Inmunología Pediátrica, Hospital Infantil Virgen del Rocío, Instituto de Biomedicina de Sevilla, RITIP, 41012 Sevilla, Spain; 3 ISGlobal, Hospital Clínic—Universitat de Barcelona, 08036 Barcelona, Spain; 4 CIBER de Enfermedades Infecciosas (CIBERINFEC), 08036 Barcelona, Spain; 5 Biomolecular Screening and Protein Technologies Unit, Centre for Genomic Regulation (CRG), The Barcelona Institute of Science and Technology, 08003 Barcelona, Spain; 6 Institut d’Investigacions Biomèdiques August Pi Sunyer (IDIBAPS), 08036 Barcelona, Spain; 7 Department of Microbiology, Immunology and Infectious Diseases, Snyder Institute for Chronic Diseases, Cumming School of Medicine, University of Calgary, Calgary, AB T2N 4N1, Canada

**Keywords:** SARS-CoV-2, antibody, antigen, COVID-19, seropositivity, cohort, children, PIMS-TS, healthcare workers

## Abstract

SARS-CoV-2 infection has become a global health problem specially exacerbated with the continuous appearance of new variants. Healthcare workers (HCW) have been one of the most affected sectors. Children have also been affected, and although infection generally presents as a mild disease, some have developed the Pediatric Inflammatory Multisystem Syndrome Temporally Associated with SARS-CoV-2 (PIMS-TS). We recruited 190 adults (HCW and cohabitants, April to June 2020) and 57 children (April 2020 to September 2021), of whom 12 developed PIMS-TS, in a hospital-based study in Spain. Using an in-house Luminex assay previously validated, antibody levels were measured against different spike and nucleocapsid SARS-CoV-2 proteins, including the receptor-binding domain (RBD) of the Alpha, Beta, Gamma, and Delta variants of concern (VoC). Seropositivity rates obtained from children and adults, respectively, were: 49.1% and 11% for IgG, 45.6% and 5.8% for IgA, and 35.1% and 7.3% for IgM. Higher antibody levels were detected in children who developed PIMS-TS compared to those who did not. Using the COVID-19 IgM/IgA ELISA (Vircell, S.L.) kit, widely implemented in Spanish hospitals, a high number of false positives and lower seroprevalences compared with the Luminex estimates were found, indicating a significantly lower specificity and sensitivity. Comparison of antibody levels against RBD-Wuhan versus RBD-VoCs indicated that the strongest positive correlations for all three isotypes were with RBD-Alpha, while the lowest correlations were with RBD-Delta for IgG, RBD-Gamma for IgM, and RBD-Beta for IgA. This study highlights the differences in antibody levels between groups with different demographic and clinical characteristics, as well as reporting the IgG, IgM, and IgA response to RBD VoC circulating at the study period.

## 1. Introduction

Since the first emergence of the severe acute respiratory syndrome coronavirus 2 (SARS-CoV-2) in 2019, significant efforts have been directed to characterize the immune response to the virus [1,2] towards understanding COVID-19 disease and immunity, and increase protection through vaccination. The main SARS-CoV-2 immunogens are the nucleocapsid (N) and spike (S) structural proteins. S is composed of two functional subunits, S1 and S2, with the receptor-binding domain (RBD) in S1 [3,4,5,6,7]. The variability of RBD epitopes is a matter of special concern, since changes in this domain in emerging variants have increased their transmission ability or immune escape from neutralizing antibodies [8,9,10,11,12]. These new variants have been named as variants of concern (VoC) by the World Health Organization (WHO): Alpha -B.1.1.7-, Beta -B.1.351-, Gamma -P.1-, Delta -B.1.617.2-, and Omicron -B.1.1.529- [8].

Healthcare workers (HCW) have been one of the most affected groups by the COVID-19 pandemic, as they are on the front line of the fight against the disease [13,14]. Many studies have addressed seroprevalence in HCW indicating a high level of exposure [15]. At the population level, the elderly are a higher risk of poor outcomes upon COVID-19, including severe disease, hospitalization, and increased mortality [16]. Conversely, most children experience a mild disease or show no symptoms [17], being mainly studied because of their capacity to transmit the virus while not being diagnosed [18,19]. Importantly, a small percentage of children may develop a new clinical syndrome named Paediatric Inflammatory Multisystem Syndrome Temporally Associated with SARS-CoV-2 (PIMS-TS) [20]. The majority of children with PIMS-TS have a history of SARS-CoV-2 infection in the preceding weeks and commonly present with fever, severe gastrointestinal symptoms, cardiovascular shock, hyperinflammation, and multisystem involvement. The immunological features of PIMS-TS involve perturbations in the immune responses, which can be reflected in antibody levels [21,22,23].

In this study, our purpose was to measure SARS-CoV-2 antibody responses using a multiplex Luminex assay in a cohort of children patients with symptoms compatible with COVID-19, as well as in a cohort of HCW and their household contacts, in a large tertiary pediatric hospital in Sevilla, Spain, in order to relate the outcome to the clinical data available. In addition, we aimed to compare the Luminex data with the results of a commercial COVID-19 ELISA IgM/IgA kit (Vircell, S.L.) frequently used in the hospital setting for diagnostic purposes, to assess the concordance between the two assays.

## 2. Materials and Methods

### 2.1. Study Design and Participants

This study was designed to determine the seroprevalence of SARS-CoV-2 antibodies in children, HCW, and their family contacts, in a cohort recruited at the Hospital Universitario Virgen del Rocío (HUVR) in Sevilla, Spain. We enrolled a total of 247 individuals who represented dissimilar clinical manifestations of SARS-CoV-2 infection and distinct age groups, based on the following criteria: (1) children < 16 years old attending the participating center with either clinical symptoms compatible with a possible SARS-CoV-2 infection (*n* = 41) or close contact with a patient diagnosed with SARS-CoV-2 infection by polymerase chain reaction (PCR) and/or antigen testing (*n* = 16), and in whom the need to take a blood sample was anticipated based on medical reasons; and (2) adults (*n* = 190) consisting of HCW (*n* = 95) from HUVR, and their family contacts (*n* = 95) who agreed to participate in the study. All participants (children and adults) had no previous history of SARS-CoV-2 vaccination before enrollment.

We measured the levels of antibodies to SARS-CoV-2 antigens in blood samples collected at baseline in children (1 April 2020 to 8 September 2021) and adults (1 April to 30 April 2020) and at a follow-up visit in adults (1 June to 30 June 2020). Three time periods of infection were defined according to predominant circulating SARS-CoV-2 variants: 1 April–31 December 2020 (Wuhan and 20E (EU1) variant); 1 January–15 June 2021 (Alpha, Beta and Gamma variants), and 15 June–8 September 2021 (Alpha, Beta, Gamma and Delta variants) [24].

Data on clinical symptoms and SARS-CoV-2 reverse transcriptase PCR (RT-PCR) or rapid antigen test results were collected prospectively from participants through questionnaires. The study was approved by the HUVR Ethics Committee and written informed consent was obtained from parents or legal guardians of participating children and from HCW and family contacts prior to blood sampling.

### 2.2. Antibody Measurements

Antibody levels were analyzed in plasma samples using two serological techniques: the COVID-19 ELISA IgG and IgM/A kits (Vircell, S.L.), and three in-house Luminex immunoassays developed to measure IgG, IgA and IgM. The COVID-19 ELISA IgG and IgM/A assays were performed manually according to the manufacturer’s instructions. The test uses recombinant SARS-CoV-2 antigens from both S and N proteins adsorbed on a solid surface. After incubation of enzyme-linked secondary antibodies in contact with a substrate, quantification was performed in a spectrophotometer. Results were expressed as OD (optical density).

The Luminex assays to measure IgA, IgG, and IgM levels (median fluorescence intensity, MFI) to different SARS-CoV-2 antigens were performed as previously described [6,25,26,27,28]. The panel of antigens included: the full-length N (N FL) protein, the C-terminal region of N (N CT), the full-length S, the subunits 1 and 2 from S (S1 and S2), and RBD located in S1, from the Wuhan strain. We also included RBDs from the Alpha, Beta, Gamma, and Delta VoCs. For the IgM and IgA measurements, samples were pre-treated with anti-human IgG (Gullsorb) at 1:10 dilution, to avoid IgG interferences. The antigen-coupled microspheres were added to a 384-well Clear^®^ flat bottom plate (Greiner Bio-One, Frickenhausen, Germany) in multiplex (2000 microspheres per analyte per well) in a volume of 90 μL of Luminex Buffer (1% BSA, 0.05% Tween 20, 0.05% sodium azide in PBS) using a 384 channels Integra Viaflo semi-automatic device. Positive and negative controls were added to each assay plate for quality assurance and control. Positive controls consisted of two hyperimmune pools (one for IgG and another one, Gullsorb pre-treated, for IgA and IgM) tested in serial dilutions. A total of 128 pre-pandemic samples were used as negative controls to establish the seropositivity thresholds, and technical blanks consisting of Luminex Buffer and microspheres without samples were added to control for non-specific (background) signal. Test samples and negative controls were tested at 1/500 dilution.

The 384-well plates with samples and beads were protected from light and incubated for 1h at room temperature in agitation at 900 rpm. Then, plates were washed 3 times with 200 μL/well of PBST (0.05% Tween 20 in PBS) using a BioTek 405 TS automatic washer, and 25 μL of goat anti-human IgG phycoerythrin (PE) at 1:400, goat anti-human IgA-PE at 1:200, or goat anti-human IgM-PE at 1:200 in Luminex buffer were added to each well, respectively, and incubated for 30 min at RT in agitation at 900 rpm. Next, plates were washed with PBST as before, and 80 μL of Luminex Buffer was added to each well to resuspend the microspheres and acquire them on a Flexmap 3D^®^ reader (at least 50 microspheres per analyte per well). MFI was reported for each antigen-antibody pair.

### 2.3. Data Analysis

Antibody MFIs obtained by Luminex measurements were log_10_-transformed and assay positivity cutoffs specific for each Ig isotype and antigen pair were calculated as 10 to the mean plus 3 standard deviations (SD) of log_10_-transformed MFI of 128 pre-pandemic controls. Results were defined as undetermined when the MFI levels for a given isotype-antigen pair were between the positivity threshold and an upper limit defined as 10 to the mean plus 4.5 SD of the log_10_-transformed MFIs of the pre-pandemic controls, and no other isotype-antigen combination was above the positivity cutoff, and the participant did not have any previous evidence of seropositivity or RT-PCR positivity. For the ELISA data, the cutoff values used were those established by the manufacturer.

Box plots were performed to visualize antibody levels in groups with different demographic and clinical characteristics. Groups were compared using the Wilcoxon Rank Sum test. Heatmaps with hierarchical clustering were performed to evaluate the patterns of antibody levels by clinical and demographic characteristics.

The Spearman rank test was used to assess the correlations between antibody levels to RDB Wuhan and RBD from VoCs, and the correlation coefficient (rho) and *p* values were reported. A *p*-value < 0.05 was considered statistically significant. All analyses were performed with R software (https://www.R-project.org/), version 4.1.2 (November 2021, accessed on 17 January 2022).

## 3. Results

### 3.1. Demographic and Clinical Characteristics of Study Participants

Demographic and clinical characteristics of the study participants are shown in Table 1. Median ages of the participating children and adults were 10 (IQR 3.25–12.75) and 44 (37–54) years, respectively. Twenty-three (43%) of 53 children with a SARS-CoV-2 RT-PCR test performed had a positive result. Forty-one (72%) children presented with clinical symptoms compatible with SARS-CoV-2 infection with a median date of symptoms onset prior to serological testing of 9 days (IQR 6–11 days). More than half (58%) of the 57 children were inpatients, including 12 (21%) cases with PIMS-TS. Serological testing in children was distributed in the following time periods: period 1 (*n* = 18, 32%), period 2 (*n* = 24, 42%), and period 3 (*n* = 15, 26%).

Despite the fact that 35 (29%) adult participants reported clinical symptoms in the previous month prior to serological testing, there was only one (1%) confirmed positive case according to the RT-PCR results.

### 3.2. Seropositivity by Luminex

Seropositivity rates in the study population (children and adults) assessed by Luminex are shown in Table 2 (see raw data in Supplementary Dataset S1). Seropositivities in pediatric patients were 49.1% for IgG, 45.6% for IgA, and 35.1% for IgM. Seropositivities for adults at baseline vs. timepoint 1 were: IgG (11% vs. 10%), IgA (5.7% vs. 4.4%), and IgM (7.3% vs. 3.9). Seropositivity rates for each isotype–RBD pair ranged as follows in children: (i) IgG (33.1% (RBD Beta)–43.3% (RBD Wuhan); *p* = 0.89), (ii) IgA (5.3% (RBD Beta)–33.3% (RBD Wuhan and Alpha), *p* = 0.0001), and (iii) IgM (1.8% (RBD Beta and Gamma)–24.6% (RBD Delta); *p* = 0.0003) (Table 3). Seropositivity rates were <5% for all isotype–RBD pairs in timepoints 0 and 1, with no significant differences among them.

### 3.3. Factors Affecting Antibody Responses

Differences in the antibody levels of the participants according to symptoms, RT-PCR results, sex, and children hospitalization, were assessed. Symptomatic children had significantly higher IgM levels against all tested antigens compared to asymptomatic children (*p* < 0.05 for N CT, N FL, RBD Beta, RBD Gamma, and S1: and *p* < 0.01 for RBD, RBD Alpha, RBD Delta, S, and S2) (Supplementary Figure S1). IgG levels were also more elevated against the majority of tested antigens in the symptomatic children (*p* < 0.05 for N FL, RBD, RBD Alpha, RBD Beta, RBD Delta, RBD Gamma, S, and S2). Children with a positive RT-PCR showed significantly higher antibody levels compared to children with a negative RT-PCR for the following isotype-antigen pairs: (i) IgG and IgA (*p* < 0.05) and IgM (*p* < 0.01) for RBD, RBD Alpha and RBD Delta, (ii) IgG, IgA and IgM (*p* < 0.05) for N FL, (iii) IgG (*p* < 0.05) and IgM (*p* < 0.01) for S, (iv) IgG and IgM (*p* < 0.05) for S2, (v) IgM (*p* < 0.05) for N CT and S1 (Supplementary Figures S1 and S2).

Inpatient children had higher IgG, IgA, and IgM levels to several antigens compared to outpatient children as follows: N FL, RBD, RBD Alpha, RBD Beta, RBD Delta, RBD Gamma, S1 (*p* < 0.05) and S and S2 (*p* < 0.01) for IgG; RBD, RBD Delta, S1 and S2 (*p* < 0.05) for IgA; and N FL, RBD Alpha, S, S1 and S2 (*p* < 0.05) and RBD and RBD Delta (*p* < 0.01) for IgM (Supplementary Figure S3). There was a trend of higher IgM levels against some antigens in female compared to male children, but differences were not significant (Supplementary Figure S4). In the case of adults, the majority of symptomatic participants were seronegative and, therefore, symptoms were likely due to reasons other than COVID-19. There were also no differences by sex, and no further clinical data could be obtained for additional analyses.

**Figure 1:**
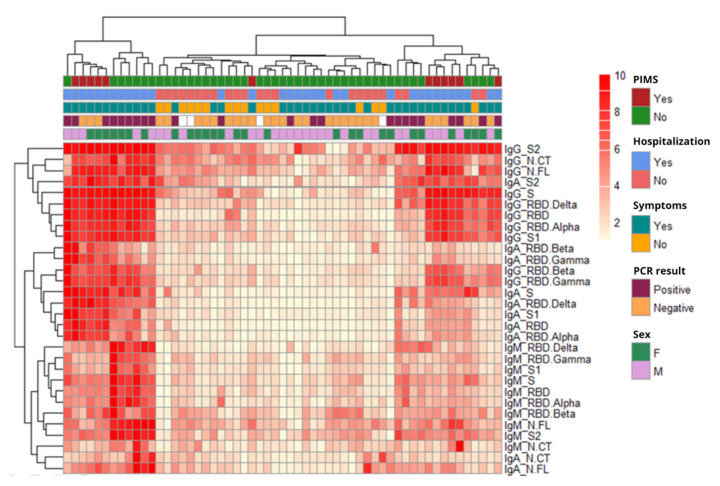
Heatmap with hierarchical clustering showing patterns of antibody responses (log_10_ MFI) in children according to clinical and demographic data. White spaces represent missing data.

We evaluated the patterns of antibody responses (log_10_ MFI) in children in relation to demographic, clinical, and microbiological data using heatmap analysis with hierarchical clustering (Figure 1). Considering all antibody responses together, patterns were very heterogeneous among participants, but they corroborated the observations described above. Specifically, lower levels of antibodies were seen in children without symptoms and non-hospitalized, and higher antibody levels were observed in children with a positive RT-PCR or with PIMS-TS. Children diagnosed with PIMS-TS clustered into two different groups, one with a predominant IgG signature, and another one with IgG plus IgA responses.

Most of the antigens clustered by isotype, and the IgM and IgA isotypes clustered closer with each other than with IgG, except for IgA S2 that clustered with IgG. Moreover, the RBD Wuhan clustered closer to the Alpha RBD than to the rest of the VoCs. No noteworthy clusters were found in the analysis of adult data, probably due to the amount of missing RT-PCR results and symptom reports (Supplementary Figure S5).

### 3.4. Antibody Responses in Relation to PIMS-TS

**Figure 2:**
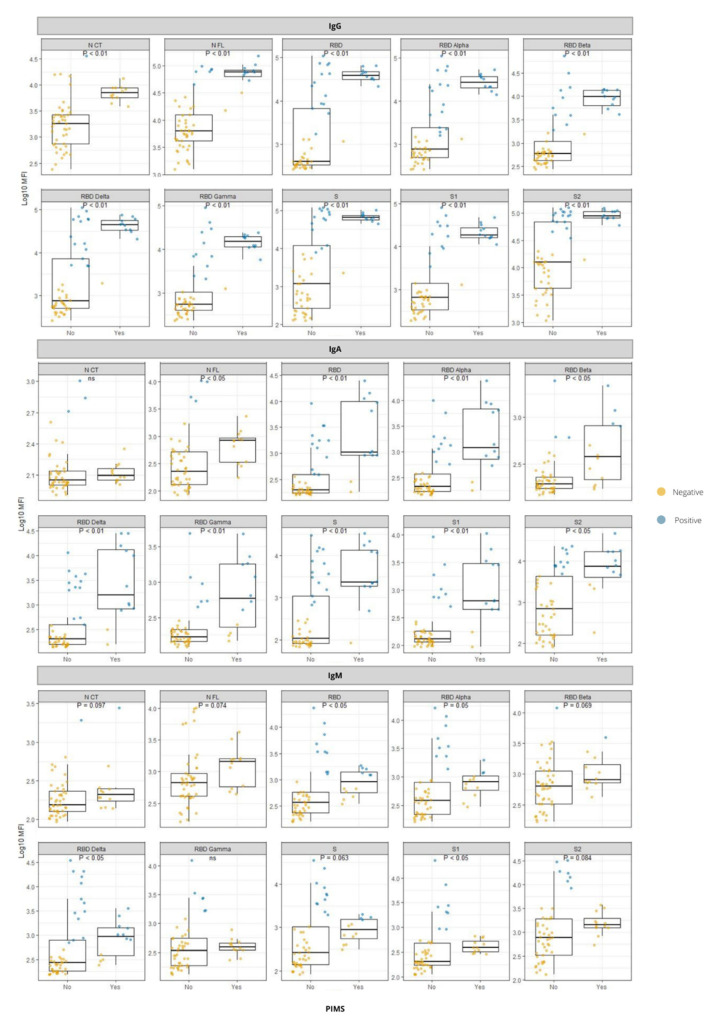
Comparison of antibody responses against different SARS-CoV-2 antigens between children with PIMS-TS and children who did not develop PIMS-TS. IgG, IgA, and IgM isotypes to different antigens tested: N CT (C-terminal region of Nucleocapsid), N FL (full-length Nucleocapsid), RBD (receptor binding domain of spike), S (full-length Spike protein), S1 (subunit 1 from S), S2 (subunit 2 from S), all from Wuhan, and RBD from VoCs: RBD Alpha, RBD Beta, RBD Delta, RBD Gamma. Dots represent each individual value, the blue color indicates a seropositive participant, and the orange color shows a seronegative participant. Groups were compared using the Wilcoxon rank-sum test.

We examined antibody responses in pediatric patients diagnosed with PIMS-TS compared to those who did not have this syndrome (Figure 2). PIMS-TS-positive had significantly higher antibody levels than PIMS-TS-negative children for almost all antigen-isotype combinations including: (1) IgG and IgA levels to all tested antigens, except for IgA response against N-CT, and (2) IgM levels against RBD, RBD Alpha, RBD Delta, and S1.

### 3.5. Comparison between ELISA and Luminex Serology

The concordance found in the results obtained with the ELISA vs. the Luminex assays was poor, as shown in Supplementary Figure S6, Tables S1 and S2. The percentage of agreement varied considerably depending on the Ig isotype analyzed. For IgG, we found a positive agreement of 37.21% and a negative agreement of 71.66%. The percentages were very low for IgA and IgM, with a positive agreement of 10.45% and 7.92%, and a negative agreement of 23.14% and 23.18%, respectively.

### 3.6. Antibody Responses to RBD from VoCs

**Figure 3:**
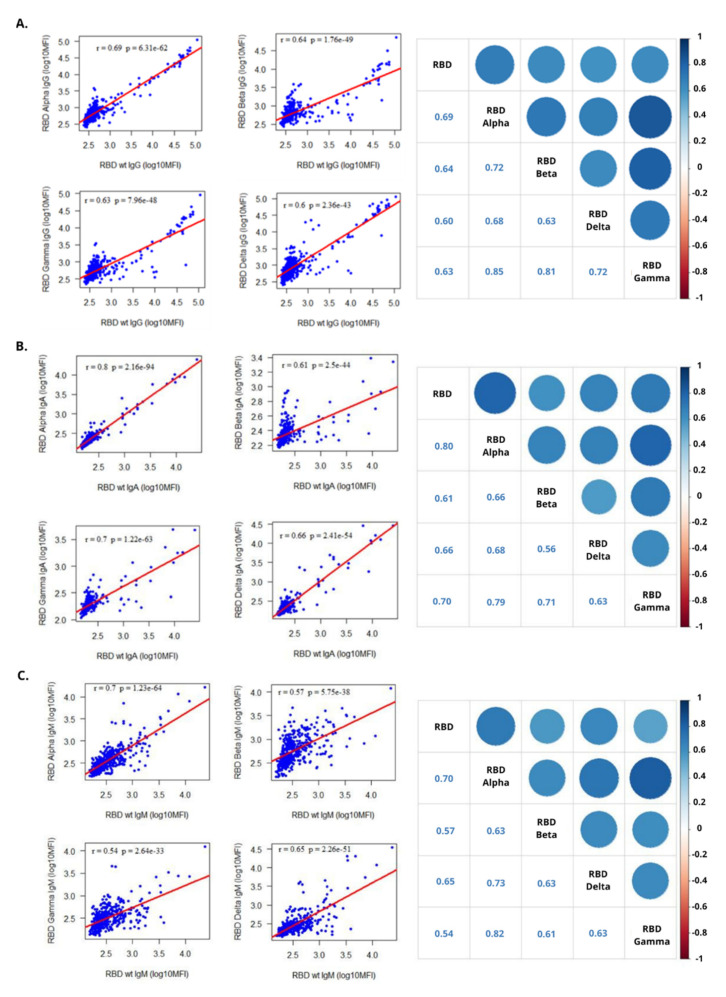
Correlation analysis using Spearman’s coefficient between Wuhan RBD and the RBDs of the different SARS-CoV-2 VoCs (Alpha, Beta, Gamma, and Delta) for each isotype: (**A**) IgG, (**B**) IgA, and (**C**) IgM.

To assess how the emerging RBD mutations presented in the different VoCs affected antibody binding, the levels of each Ig to RBD from Alpha, Beta, Gamma, and Delta variants were compared to the Wuhan RBD (Figure 3). For IgG, the RBD variant that presented a higher correlation index with the RBD Wuhan was Alpha (r = 0.69; *p* < 0.0001), followed by Beta (r = 0.64; *p* < 0.0001), Gamma (r = 0.63; *p* < 0.0001) and Delta (r = 0.60; *p* < 0.0001). For IgA, the RBD with the highest correlation index with the RBD Wuhan was again Alpha (r = 0.8; *p* < 0.0001) followed by the Gamma (r = 0.7; *p* < 0.0001), Delta (r = 0.66; *p* < 0.0001), and Beta (r = 0.61; *p* < 0.0001). For IgM, the RBD with the highest correlation index with the Wuhan RBD was also Alpha (r = 0.7; *p* < 0.0001), followed by Delta (r = 0.65; *p* < 0.0001), Beta (r = 0.57; *p* < 0.0001), and Gamma (r = 0.54; *p* < 0.0001). 

## 4. Discussion

We examined the SARS-CoV-2 seropositivity rates in a cohort of pediatric patients with symptoms compatible with COVID-19 and the close contacts to infected children, and a cohort of HCWs and cohabitants, in a hospital-based setting, by using a broad panel of SARS-CoV-2 antigens to increase sensitivity of detection. One-fifth of the HCWs were seropositive, showing a higher percentage estimate than that of the general population of Andalucia at that date (April–June 2020), and similar to that of HCWs from other hospitals [26,29,30].

Half of children (54.4%) were seropositive, and those who required hospitalization showed higher antibody levels. Consistently, previous studies have reported slightly higher levels of antibodies in hospitalized and symptomatic patients [31,32,33,34]. In addition, there was a trend for increased levels of all three Ig isotypes against some antigens in females compared to males. Previous studies have suggested that antibody responses are higher in females in cases of severe disease [35,36].

PIMS-TS is a rare but severe and potentially fatal complication in children with COVID-19, and therefore it is important to elucidate the immune mechanisms underlying it. We found a stronger antibody response in pediatric patients who developed PIMS-TS than those who did not. However, this observation could be partly related to differences in time periods from infection onset to serological study between both groups. To this regard, children with PIMS-TS generally present with clinical symptoms 2 to 6 weeks after an initial mild or unapparent SARS-CoV-2 infection that could have been initially overlooked. This type of response has been found to be common, especially for IgG, describing this syndrome as a late manifestation of the infection producing a hyperimmune response. Autoantibodies expressed against cardiac and gastrointestinal endothelial tissues have been found; however, it has not been elucidated whether these are primary mediators of the disease or are a secondary cause produced as a result of tissue damage after infection [37,38,39,40,41].

Regarding the antibody responses to RBD from VoCs, we observed that seropositivities were higher against Alpha and Delta RBDs, which is consistent with the predominant circulation of these variants during the sample collection period, with Beta and Gamma being a minority [24,42,43,44]. However, the mutations present in the RBD VoC can interfere with antibody binding and cause neutralization refractoriness [9,45,46,47]. The Alpha RBD showed the highest correlation with the RBD Wuhan for all isotypes, probably because it is the VoC that genetically most closely resembles the wild type compared to the other three. While the lowest correlations were with RBD Delta for IgG, RBD Beta for IgA, and RBD Gamma for IgM; these VoC carry more mutations relative to the Wuhan RBD and at more specific binding sites related to immune evasion [48,49,50].

Finally, in all cases, a reliable and effective serological diagnosis of SARS-CoV-2 is very important in a hospital setting; thus, we compared a commonly used commercial kit with our in-house method that has shown excellent performance. In our comparison of the Luminex assay with the COVID-19 ELISA (Vircell, S.L.), the percentage of agreement was quite low, especially for the IgA and IgM isotypes. As shown in previous studies [51] using the Vircell ELISA, a high number of false positives was obtained in comparison with the more sensitive and specific Luminex technique.

The sample size of the children cohort was limited because we did not consider it necessary to take blood samples in the majority of children with SARS-CoV-2 infection attending the hospital emergency department during the study period as they were generally asymptomatic or had only mild illness. Reduced sample size could have undermined the ability to detect statistically significant differences.

In conclusion, we used a reliable assay for the study of seropositivity in a cohort of pediatric patients and another cohort of healthcare personnel and cohabitants. We obtained accurate antibody profiles in different demographic groups and with different clinical characteristics. We found differences in antibody levels depending on whether the pediatric patients had been hospitalized or had developed PIMS-TS. Reporting new features in this type of syndrome is very important as it can cause severe consequences in children. In turn, the study of antibody levels and seroprevalence including VoC antigens is relevant to elucidate how mutations carried by the new viral variants may affect antibody binding and thus cause escape of the immune response, an approach that is necessary for the development and preparation of second-generation vaccines.

## Figures and Tables

**Table 1:** Demographic and clinical characteristics of study participants.

	Children	Adults	
Timepoint	0 (baseline)	0	1
*N*	57	190	172
Median age, years (IQR)	10 (3.25–12.75)	44 (37–54)	NA
Sex (M/F)	1.04	1.06	0.81
Symptoms *, *n* (%)	41 (72)	35 ^†^ (29)	NA
Median days from symptoms onset to serological study (IQR)	9 (6–11)	NS	NA
Positive SARS-CoV-2 RT-PCR, *n* (%)	23 (43) ^§^	1 (1) ^‡^	NA
Hospitalized, *n* (%)	33 (58)	NA	NA
PIMS, *n* (%)	12 (21)	NA	NA
Time periods of recruitment ^ǂ^			
1. *n* (%)	18 (32)	190 (100)	172 (100)
2. *n* (%)	24 (42)		
3. *n* (%)	15 (26)		

* Fever and/or respiratory symptoms. ^†^ Data available from 121 adults. ^§^ Performed in 53 children. ^‡^ Performed in 107 adults. NA = not applicable. NS = not specified. PIMS = paediatric multisystem inflammatory syndrome. ^ǂ^ Time periods according to predominant circulating SARS-CoV-2 variants: (1) 1 April–31 December 2020 (Wuhan and 20E (EU1) variants); (2) 1 January–15 June 2021 (Alpha, Beta and Gamma variants); and (3) 15 June–8 September 2021 (Alpha, Beta, Gamma, and Delta variants).

**Table 2:** Seroprevalence in study participants by isotype and isotype-antigen combination measured by Luminex.

	Children (*n* = 57)					Adults Timepoint 0 (*n* = 190)					Adults Timepoint 1 (*n* = 172)				
	Positive		Undeter-mined		Positive + Undeter.	Positive		Undeter-mined		Positive + Undeter.	Positive		Undeter-mined		Positive + Undeter.
	*n*	%	*n*	%	%	*n*	%	*n*	%	%	*n*	%	*n*	%	%
**Overall**															
	31	54.39	7	12.28	66.67	37	19.37	28	14.66	34.03	32	18.60	20	11.63	30.23
**By isotype**															
IgG	28	49.12	3	5.26	54.39	21	10.99	11	5.76	16.75	18	9.94	5	2.76	12.71
IgA	26	45.61	5	8.77	54.39	11	5.76	15	7.85	13.61	8	4.42	12	6.63	11.05
IgM	20	35.09	4	7.02	42.11	14	7.33	14	7.33	14.66	7	3.87	12	6.63	10.5
**By isotype-antigen combination**															
IgG N CT	0	0	1	1.75	1.75	0	0	2	1.05	1.05	0	0	1	0.55	0.55
IgG N FL	3	5.26	13	22.81	28.07	2	1.05	8	4.19	5.24	2	1.1	4	2.21	3.31
IgG RBD Wuhan	24	42.11	1	1.75	43.86	9	4.71	4	2.09	6.81	8	4.42	1	0.55	4.97
IgG S	22	38.6	3	5.26	43.86	4	2.09	4	2.09	4.19	3	1.66	4	2.21	3.87
IgG S1	21	36.84	1	1.75	38.6	6	3.14	3	1.57	4.71	5	2.76	3	1.66	4.42
IgG S2	19	33.33	8	14.04	47.37	2	1.05	7	3.66	4.71	4	2.21	1	0.55	2.76
IgA N CT	2	3.51	1	1.75	5.26	3	1.57	2	1.05	2.62	0	0	3	1.66	1.66
IgA N FL	2	3.51	2	3.51	7.02	2	1.05	4	2.09	3.14	0	0	2	1.1	1.1
IgA RBD Wuhan	19	33.33	3	5.26	38.6	2	1.05	1	0.52	1.57	2	1.1	1	0.55	1.66
IgA S	24	42.11	1	1.75	43.86	4	2.09	6	3.14	5.24	4	2.21	3	1.66	3.87
IgA S1	15	26.32	3	5.26	31.58	3	1.57	0	0	1.57	0	0	3	1.66	1.66
IgA S2	7	12.28	13	22.81	35.09	1	0.52	6	3.14	3.66	0	0	5	2.76	2.76
IgM N CT	0	0	2	3.51	3.51	0	0	4	2.09	2.09	0	0	2	1.1	1.1
IgM N FL	0	0	0	0	0	0	0	1	0.52	0.52	0	0	0	0	0
IgM RBD Wuhan	7	12.28	9	15.79	28.07	2	1.05	7	3.66	4.71	0	0	6	3.31	3.31
IgM S	9	15.79	6	10.53	26.32	1	0.52	6	3.14	3.66	0	0	4	2.21	2.21
IgM S1	6	10.53	2	3.51	14.04	0	0	3	1.57	1.57	0	0	1	0.55	0.55
IgM S2	2	3.51	5	8.77	12.28	2	3.51	5	8.77	12.28	0	0	2	1.1	1.1

**Table 3:** Seroprevalence in study participants by isotype-RBD combination, including VoC RBDs (Alpha, Beta, Gamma and Delta), measured by Luminex.

	Children (*n* = 57)						Adults Timepoint 0 (*n* = 190)					Adults Timepoint 1 (*n* = 172)						
	Positive		Undeter-mined		Positive + Undeter.	*p* value *	Positive		Undeter-mined		Positive + Undeter.	*p* value *	Positive		Undeter-mined		Positive + Undeter	*p* value *
	*n*	%	*n*	%	%		*n*	%	*n*	%	%		*n*	%	*n*	%	%	
IgG RBD						0.89						0.35						0.35
IgG RBD Wuhan	24	42.11	1	1.75	43.86		9	4.71	4	2.09	6.81		8	4.42	1	0.55	4.97	
IgG RBD Alpha	22	38.6	4	7.02	45.61		8	4.19	8	4.19	8.38		8	4.42	4	2.21	6.63	
IgG RBD Beta	19	33.33	2	3.51	36.84		3	1.57	4	2.09	3.66		4	2.21	1	0.55	2.76	
IgG RBD Gamma	21	36.84	1	1.75	38.6		4	2.09	5	2.62	4.71		3	1.66	4	2.21	3.87	
IgG RBD Delta	23	40.35	3	5.26	45.61		6	3.14	6	3.14	6.28		4	2.21	6	3.31	5.52	
IgA RBD						0.0001						NA						NA
IgA RBD Wuhan	19	33.33	3	5.26	38.6		2	1.05	1	0.52	1.57		2	1.1	1	0.55	1.66	
IgA RBD Alpha	19	33.33	1	1.75	35.09		3	1.57	2	1.05	2.62		2	1.1	1	0.55	1.66	
IgA RBD Beta	3	5.26	4	7.02	12.28		0	0	3	1.57	1.57		0	0	4	2.21	2.21	
IgA RBD Gamma	9	15.79	5	8.77	24.56		1	0.52	1	0.52	1.05		0	0	2	1.1	1.1	
IgA RBD Delta	21	36.84	1	1.75	38.6		2	1.05	2	1.05	2.09		2	1.1	3	1.66	2.76	
IgM RBD						0.0003						NA						NA
IgM RBD Wuhan	7	12.28	9	15.79	28.07		2	1.05	7	3.66	4.71		0	0	6	3.31	3.31	
IgM RBD Alpha	8	14.04	4	7.02	21.05		3	1.57	4	2.09	3.66		2	1.1	1	0.55	1.66	
IgM RBD Beta	1	1.75	1	1.75	3.51		0	0	2	1.05	1.05		0	0	0	0	0	
IgM RBD Gamma	1	1.75	5	8.77	10.53		1	0.52	1	0.52	1.05		1	0.55	0	0	0.55	
IgM RBD Delta	14	24.56	7	12.28	36.84		3	1.57	10	5.24	6.81		1	0.55	6	3.31	3.87	

* For comparison of seropositivity (positive results) rates among isotype-RBD pairs. NA = Not applicable.

## Data Availability

Not applicable.

## Supplementary Materials

The following supporting information can be downloaded at: https://www.mdpi.com/article/10.3390/v14092039/s1, Supplementary Figure S1. Comparison of antibody responses against different SARS-CoV-2 antigens (N CT, N FL, RBD, S, S1, S2, and RBD from VoC: RBD Alpha, RBD Beta, RBD Delta, RBD Gamma) between children with symptoms and children who did not develop symptoms; Supplementary Figure S2. Comparison of antibody responses against different SARS-CoV-2 antigens (N CT, N FL, RBD, S, S1, S2, RBD from VoC: RBD Alpha, RBD Beta, RBD Delta, RBD Gamma) between children with a RT-PCR+ and those with a RT-PCR-; Supplementary Figure S3. Comparison of antibody responses against different SARS-CoV-2 antigens (N CT, N FL, RBD, S, S1, S2, RBD from VoC: RBD Alpha, RBD Beta, RBD Delta, RBD Gamma) between children who required hospitalization and those who did not; Supplementary Figure S4. Comparison of antibody responses against different SARS-CoV-2 antigens (N CT, N FL, RBD, S, S1, S2, RBD from VoC: RBD Alpha, RBD Beta, RBD Delta, RBD Gamma) between female and male children; Supplementary Figure S5. Heatmaps with hierarchical clustering for adult data including antibody levels (log_10_ MFI) and clinical and demographic data at timepoint 0 (left) and timepoint 1 (right); Supplementary Figure S6. Classification and comparison of ELISA and Luminex results for IgG (A), IgA (B) and IgM (C) isotypes; Supplementary Table S1. Classification of samples by ELISA (E) and Luminex (L) assays for IgG, IgM and IgA; Supplementary Table S2. Percentage of agreement between ELISA and Luminex results for IgG, IgA and IgM; Supplementary Dataset S1. Luminex Mean Fluorescent Intensities (MFI) of samples.
