# Supplementary material for: SARS-CoV-2 Seroprevalence Study in Pediatric Patients and Health Care Workers Using Multiplex Antibody Immunoassays
Source: Viruses. 2022 Sep 14;14(9):2039. doi: 10.3390/v14092039 (PMC9502584; doi:10.3390/v14092039)
Supplement: Supplementary file 1 [file viruses-14-02039-s001.zip › Supplementary Figures and Tables.pdf]

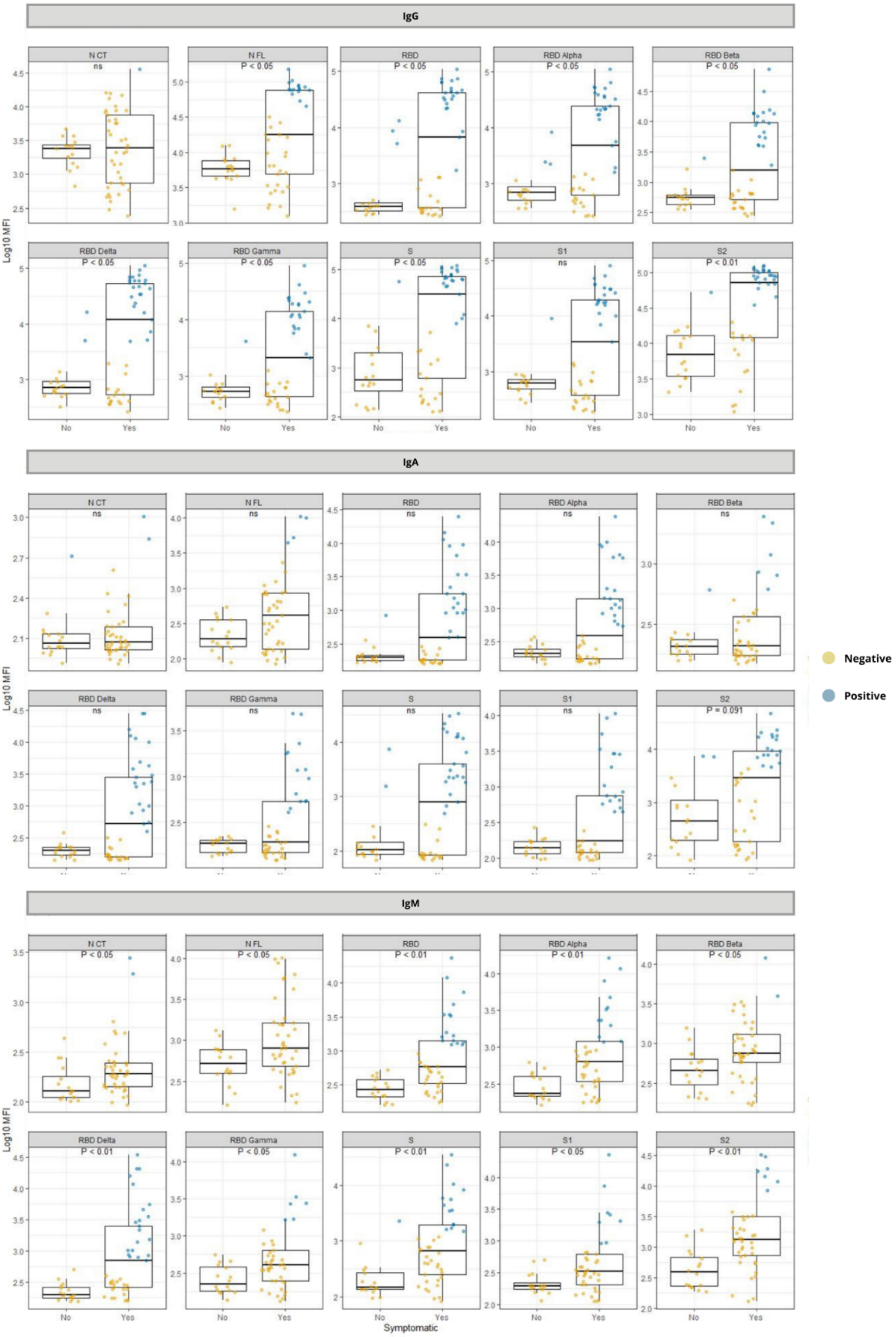

Supplementary Figure S1. Comparison of antibody responses against different SARS-CoV-2 antigens (N CT, N FL, RBD, S, S1 y S2 y RBD from VoC: RBD alpha, RBD beta, RBD delta, RBD gamma) between children with symptoms and children who did not develop symptoms. IgG (A), IgA(B) and IgM (C) isotypes. Dots represent each individual value, blue color indicates a seropositive participant and the orange color shows a seronegative participant. Groups were compared using the Wilcoxon rank-sum test.

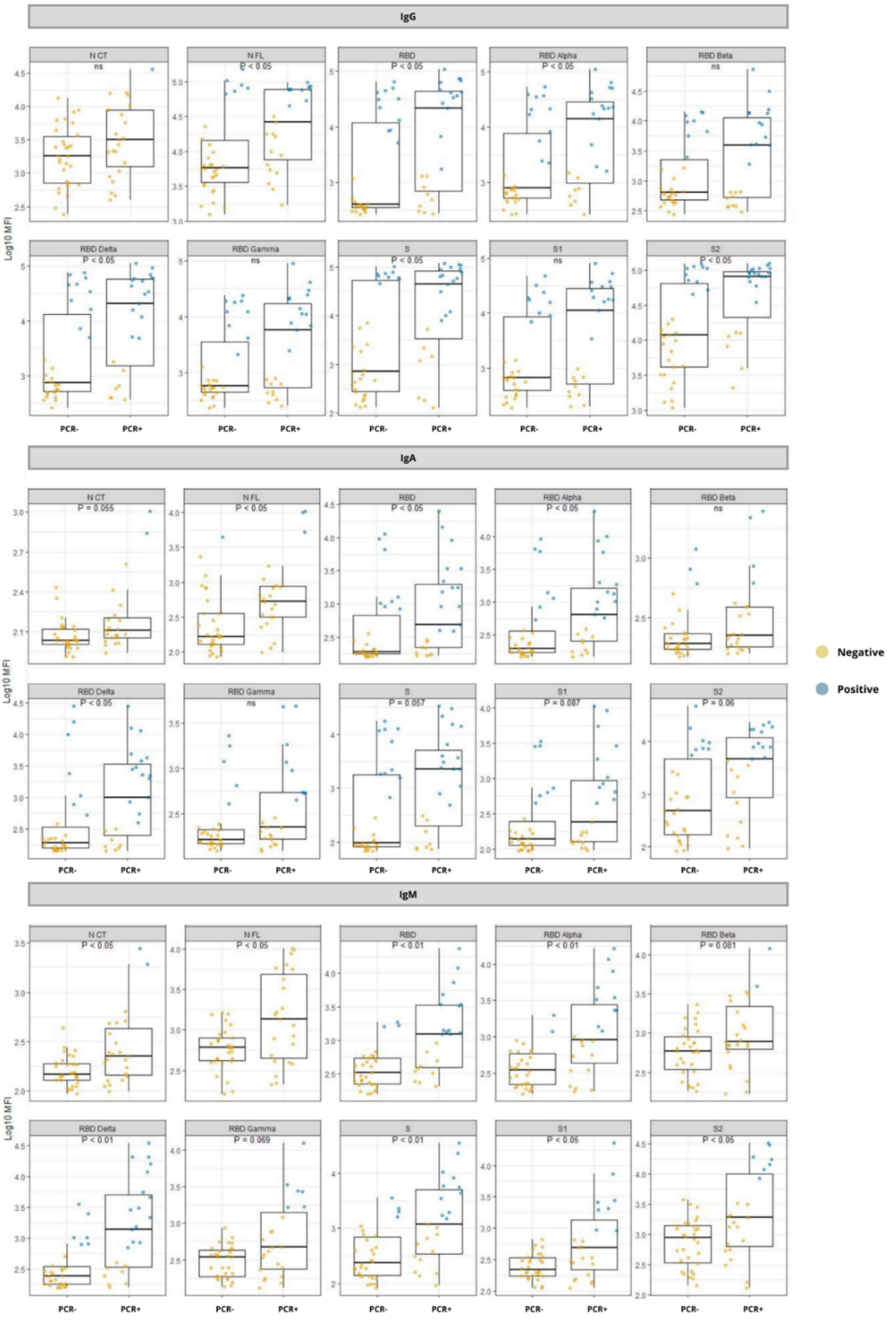

Supplementary Figure S2. Comparison of antibody responses against different SARS-CoV-2 antigens (N CT, N FL, RBD, S, S1 y S2 y RBD from VoC: RBD alpha, RBD beta, RBD delta, RBD gamma) between children with a PCR+ and those with a PCR-. IgG (A), IgA(B) and IgM (C) isotypes. Dots represent each individual value, blue color indicates a seropositive participant and the orange color shows a seronegative participant. Groups were compared using the Wilcoxon rank-sum test.

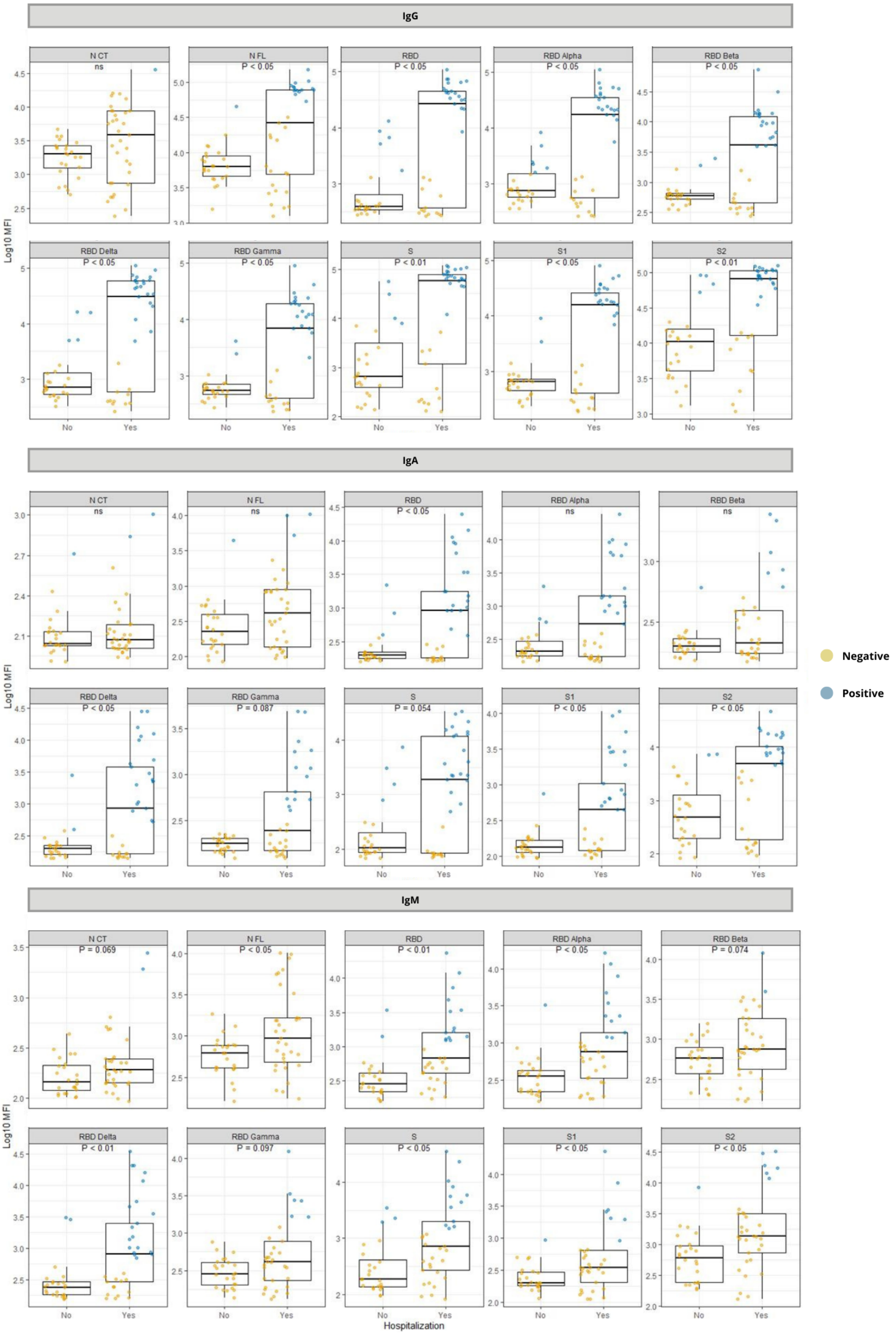

Supplementary Figure S3. Comparison of antibody responses against different SARS-CoV-2 antigens (N CT, N FL, RBD, S, S1 y S2 y RBD from VoC: RBD alpha, RBD beta, RBD delta, RBD gamma) between children who required hospitalization and those who did not. IgG (A), IgA(B) and IgM (C) isotypes. Dots represent each individual value, blue color indicates a seropositive participant and the orange color shows a seronegative participant. Groups were compared using the Wilcoxon rank-sum test.

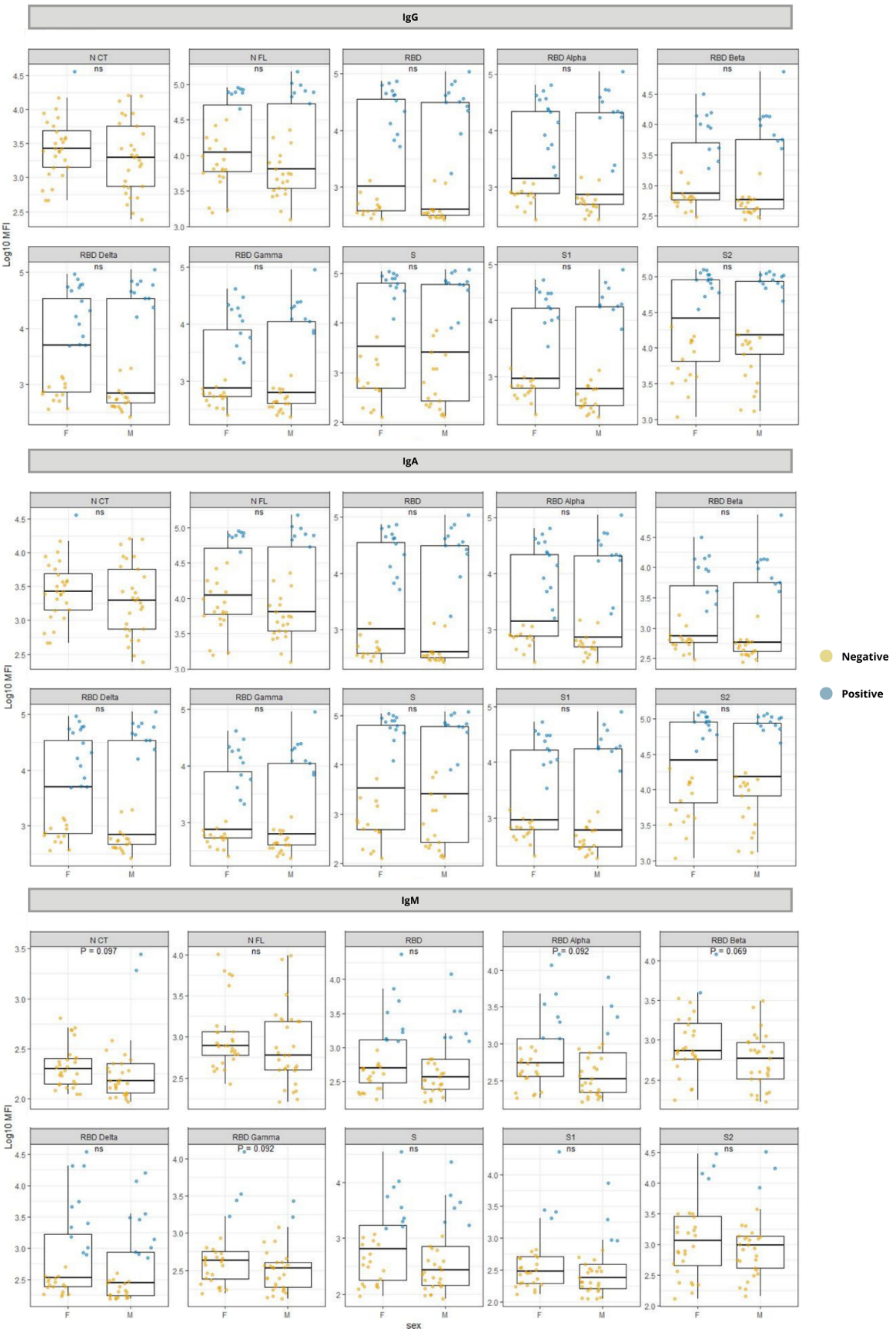

Supplementary Figure S4. Comparison of antibody responses against different SARS-CoV-2 antigens (N CT, N FL, RBD, S, S1 y S2 y RBD from VoC: RBD alpha, RBD beta, RBD delta, RBD gamma) between female and male children. IgG (A), IgA(B) and IgM (C) isotypes. Dots represent each individual value, blue color indicates a seropositive participant and the orange color shows a seronegative participant. Groups were compared using the Wilcoxon rank-sum test.

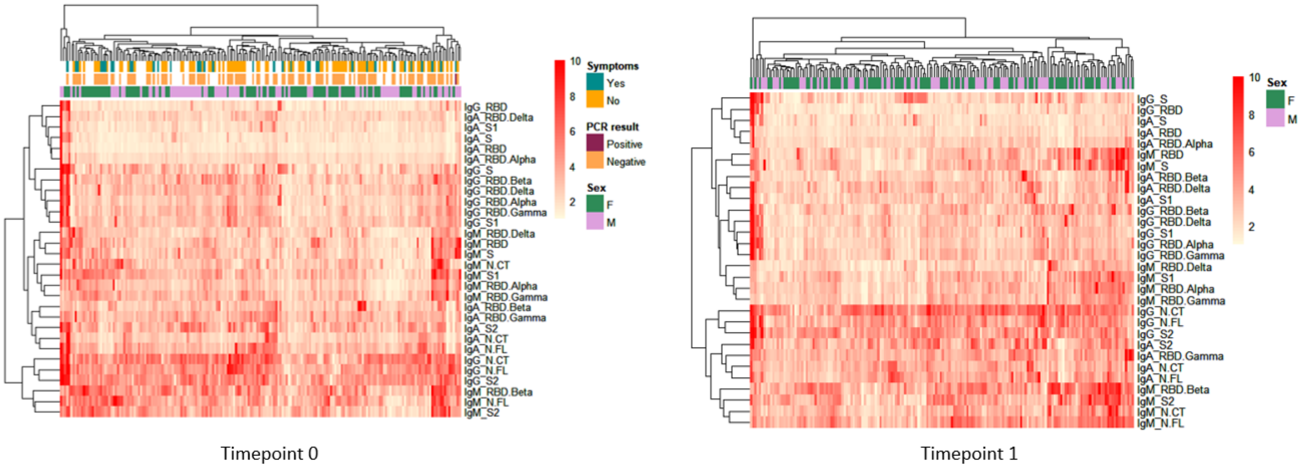

Supplementary Figure S5. Heatmaps with hierarchical clustering for adults data including antibody levels (log_{10} MFI) and clinical and demographic data at timepoint 0(left) and timepoint 1(right). White spaces represent missing data.

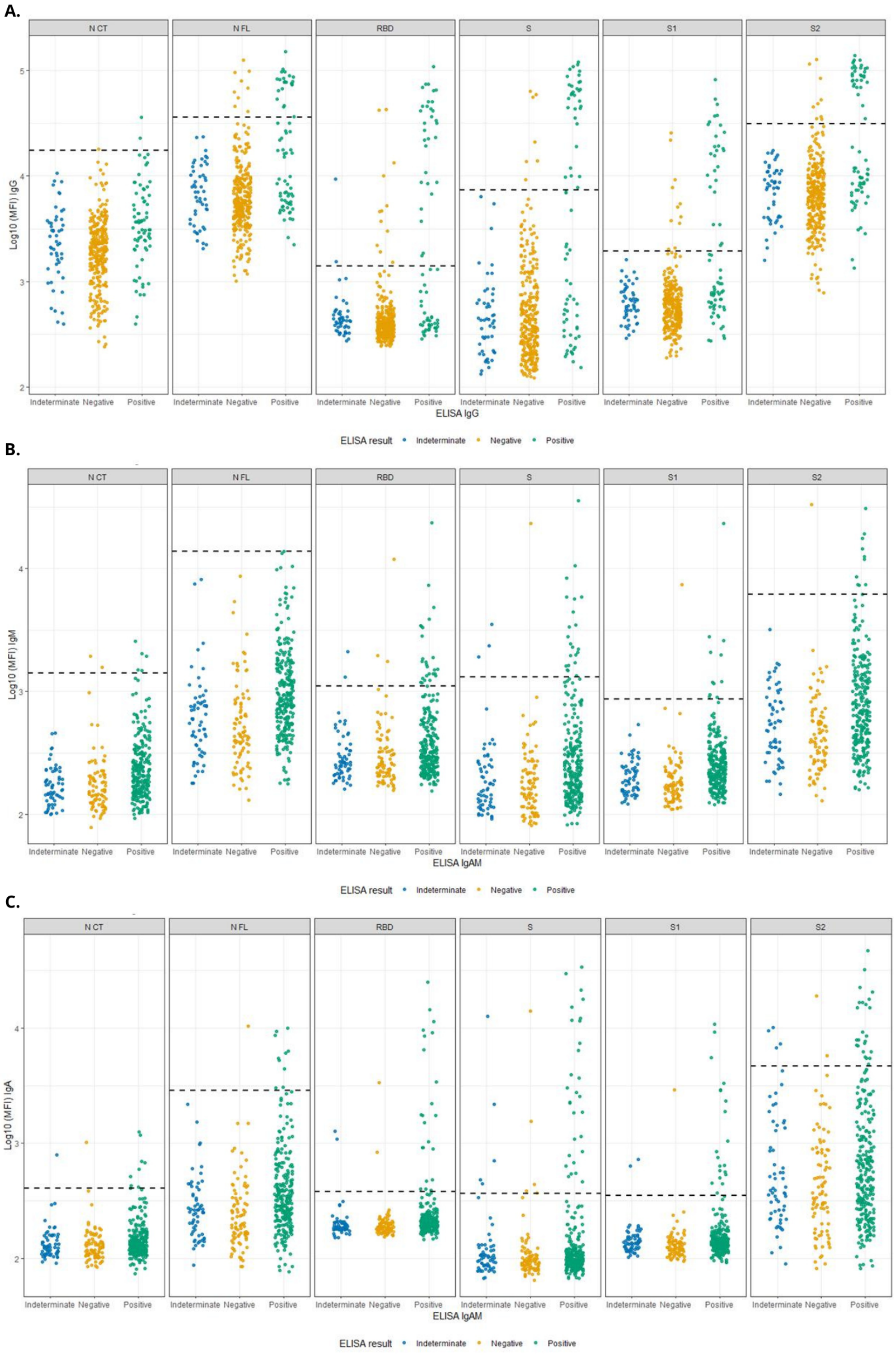

Supplementary Figure S6. Classification and comparison of ELISA and Luminex results for IgG (A), IgA(B) and IgM(C) isotypes. The X axis shows the results obtained by ELISA assay, according to the cutoffs established by the manufacturer for each isotype (Positive, in green; negative, in yellow; indeterminate, in blue). The Y axis shows the antibody level obtained by Luminex as $\log_{10}$ of the mean fluorescence intensity (MFI) and dashed line shows the seropositivity cutoff for each antigen.

Supplementary Table S1. Classification of samples by ELISA (E) and Luminex (L) assays for IgG, IgM and IgA. (+) indicates a positive result by that assay, (-) indicates a negative result and (~) indicates that the serology result was undetermined.

Isotype	L+,E+	L+,E-	L+,E~	L-,E-	L-,E~	L-,E+	L~,E~	L~,E-	L~,E+
IgG	32	17	1	268	48	32	1	8	4
IgA	28	2	4	84	52	217	3	4	17
IgM	21	1	2	86	55	224	2	3	17

*L: Luminex; E: Elisa

Supplementary Table S2. Percentage of agreement between ELISA and Luminex results for IgG, IgA and IgM. The percentages of agreement were calculated by dividing the concordant positive samples (L+ E+) or concordant negative samples (L-, E-) by the total of samples that tested positive or negative in at least one of the tests, respectively.

Isotype	Positive agreement (%)	Negative agreement (%)	Indeterminate agreement (%)
IgG	37.21	71.66	1,61
IgA	10.45	23.14	3.75
IgM	7.92	23.18	2.53
